# Bentall surgery and total arch repair with debranching of supra-aortic vessels: a case report

**DOI:** 10.1186/s43044-022-00248-y

**Published:** 2022-02-21

**Authors:** Wilfredo Luna Victoria-Medina, Carlos Quispe-Vizcarra, Miguel Rojas-Huillca, Milagros Moreno-Loaiza, W. Samir Cubas

**Affiliations:** Service of Heart Surgery, Department of Thoracic and Cardiovascular Surgery, Edgardo Rebagliati Martins National Hospital, Avenue Rebagliati 490, Jesus Maria, Lima, Peru

**Keywords:** Ascending aortic aneurysm, Aortic arch, Bentall procedure, Debranching, Anterograde cerebral perfusion, Deep hypothermic circulatory arrest

## Abstract

**Background:**

The surgical approach to pathologies of the Ascending Thoracic Aorta (ATA) that compromise aortic root and the aortic arch is currently one of the most complex interventions in the spectrum of cardiac surgery, where circulatory arrest with cerebral perfusion plays an important role for Success postoperative and patient survival.

**Case presentation:**

We present the case of a 57-year-old patient with the only history of arterial hypertension and an ATA Aneurysm that compromised segment of the aortic root up to segment 2 of the aortic arch. A successful Bentall surgery was performed, debranching supra-aortic vessels with Total Circulatory Arrest with Deep Hypothermic Cerebral Perfusion-Antegrade Bilateral.

**Conclusions:**

With the advent of new anesthetic and neuroprotection techniques, perioperative imaging protocols, advanced hemodynamic monitoring, and invaluable advances in perfusion and Extracorporeal Circulation with circulatory arrest, they have made this surgical challenge a valuable tool for today’s cardiovascular surgeon.

## Background

Surgical pathology of the Ascending Thoracic Aorta (ATA) is relatively frequent and mainly includes aneurysms, dissections, and ruptures. Ascending Thoracic Aortic Aneurysms (ATAA) top the list with an incidence of 6–10 cases/100,000 inhabitants per year and occur between the sixth and eighth decade of life with a greater predilection in men over women (2:1) [1]. Around 25% of patients with large ATAA also carry pathologies of the aortic root, aortic arch, and abdominal aorta; mostly related to hereditary non-autoimmune collagen diseases such as Marfan and Ehlder-Danlos [2].

The main surgical objective of ATAA is the restoration of the normal dimensions of the same, considering the body surface area, and preventing the risk of dissection or rupture [3]. The latter is associated with mortality greater than 90% and has led recent guidelines to propose the timely surgical treatment of ATA with maximum diameters of 45 mm in Marfan, 50 mm in bicuspid aortic valve disease, and 55 mm in patients without known comorbidities [4].

With the advent of new anesthetic and neuroprotection techniques, perioperative imaging protocols, advanced hemodynamic monitoring, and invaluable advances in perfusion and Extracorporeal Circulation (EC) with circulatory arrest, they have made this surgical challenge a valuable tool for today's cardiovascular surgeon [5].

## Case presentation

A 57-year-old woman with a history of arterial hypertension came to the emergency service due to moderate chest pain radiated to the anterior cervical region and of an oppressive type, associated with dyspnea on moderate efforts (NYHA II). This symptomatology presented insidiously for 2 months and presented a progressive course until his hospital admission. The general physical examination showed an endomorph anatomical constitution, a Body Mass Index of 37, and no marfanoid features. The cardiovascular evaluation, an aortic diastolic murmur (III/IV), and peripheral arterial pulses were identified without alterations.

**Fig. 1:**
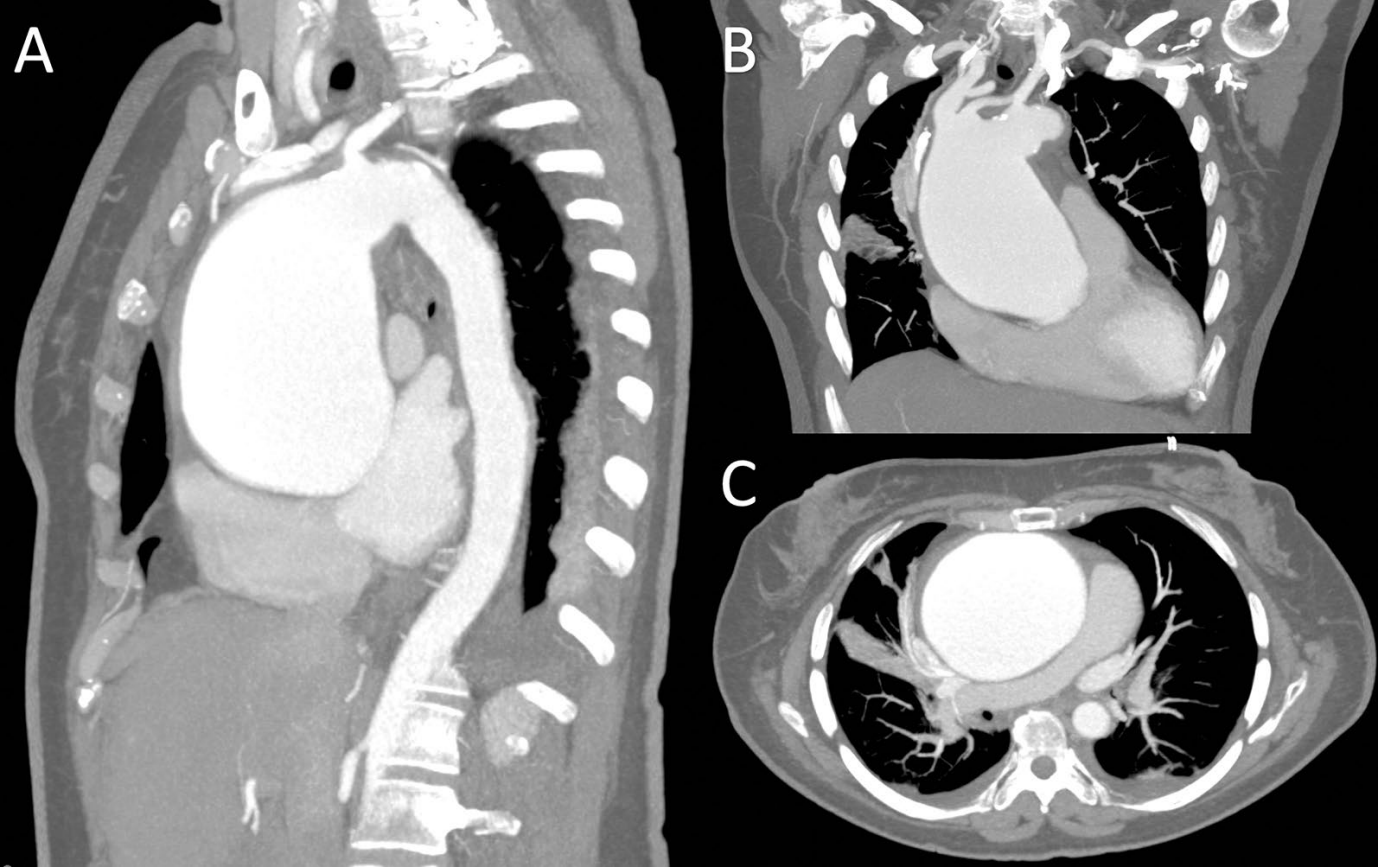
**A–C** Angiotomography with coronal, sagittal and axial view

**Fig. 2:**
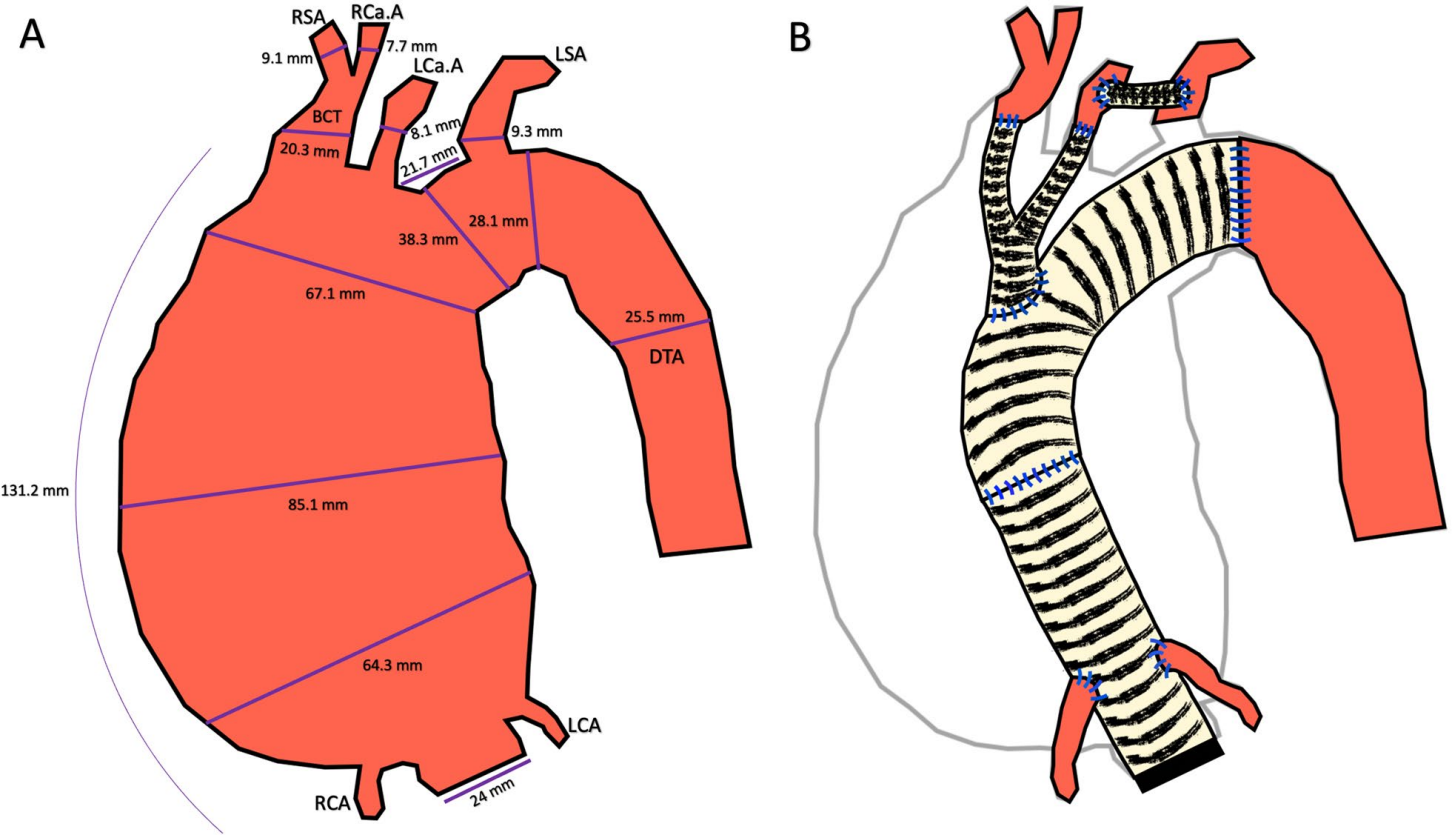
**A** Preoperative diagrammatic view: measurements of the ATAA and supra-aortic vessels. **B** Postoperative diagrammatic view: Bentall Surgery + Total Aortic Arch Replacement with Supra-Aortic Vessel Debranching. *RCA* Right Coronary Artery, *LCA* Left Coronary Artery, *BCT* Brachiocephalic-Trunk, *RSA* Right Subclavian Artery, *LSA* Left Subclavian Artery, *RCa.A* Right Carotid Artery, *LCa.A* Left Carotid Artery, *DTA* Descending Thoracic Aorta

Laboratory parameters were normal but the angiotomographic study revealed an ATAA with a diameter greater than 85 mm, which extended from the aortic sinotubular junction to zone 2 of the aortic arch (Figs. 1, 2). Likewise, the Transthoracic Echocardiogram (TEE) reported severe aortic regurgitation with an ATAA that extended into the aortic arch, and coronary angiography did not show significant coronary lesions.

Surgical repair was immediately considered with Bentall surgery, debranching of supra-aortic vessels with EC (myocardial protection, Custodiol®), and Total Circulatory Arrest with Deep Hypothermic Cerebral Perfusion-Antegrade Bilateral (TCADHCP-AB) by extension of the dacron towards the artery axillar right.

**Fig. 3:**
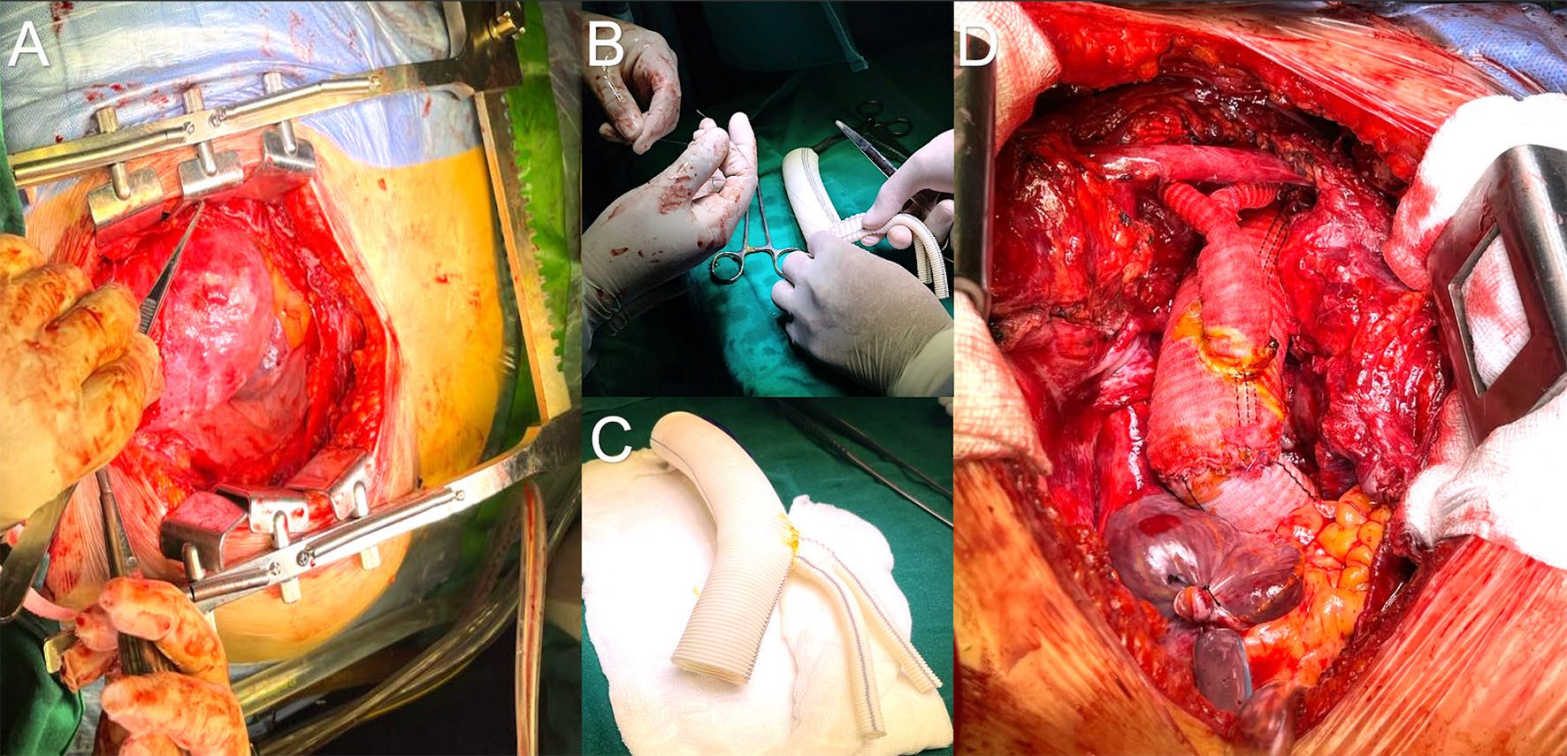
Intraoperative views **A** ATAA by median sternotomy approach. **B, C** Table construction of aortic arch graft. **D** Bentall Surgery + Total Aortic Arch Replacement with Supra-Aortic Vessel Debranching

Due to the high risk of aneurysmal rupture during the surgical opening, the onset of peripheral EC with an arterial “Y” configuration (clamped right axillary and femoral artery) was considered before median sternotomy. After opening, the aortic cross-clamp was performed and Bentall surgery was initially performed (24/26 mm valve tube), while another surgical team constructed the aortic arch graft on the table (28 mm dacron linear graft + bifurcated dacron 14 × 7 mm). Subsequently, the EC was stopped and the TCADHCP-AB (Bicarotid, Temperature 19°, 52′) was started before unclamping the right axillary arterial cannula, clamping in the Brachiocephalic Trunk (BCT), and clamping the peripheral femoral artery, to anastomosing the aortic arch graft in proximal zone 3. Once the distal aortic anastomosis was concluded, the circulatory arrest was completed and the EC (femoral and axillary “Y” unclamped) was re-entered with a cross-clamp on the aortic arch graft, to continue with the dacron Bentall-aortic arch anastomosis. The cross-clamp (188′) was removed, and the EC (246′) was completed after anastomosis of the supra-aortic vessels, 7 mm dacron-BCT, 7 mm Dacron-Left Carotid Artery (LCa.A), and bypass with interposition of dacron 7 mm between LCa.A-Left Subclavian Artery (LSA) (Fig. 3).

The stay in postoperative intensive care was relatively long due to ventilator-associated pneumonia and no evidence of neurological injury; however, the patient was discharged successfully with an indication for permanent anticoagulation and outpatient clinic visits.

## Discussion

Ascending aorta and aortic arch surgery is currently one of the most complex surgical interventions in the spectrum of cardiac surgery, where cardiac and circulatory arrest play an important role in the post-surgical success and patient survival. Our case met the main recommendation criteria of the *European Association for Cardio-Thoracic Surgery* and the *European Society for Vascular Surgery* for open treatment of TAA (high risk of retrograde dissection with ascending aorta > 45 mm, aneurysmal extension involving aortic root and extending to the aortic arch) [6].

Despite this, the open approach involves a wide variety of techniques such as circulatory arrest with cerebral perfusion, allowing us to obtain greater surgical safety with less risk of systemic and cerebral ischemia. These techniques require adequate planning and strict control of the times of ischemia, hypothermia, and cerebral perfusion.

For this reason, TCADHCP-AB was used through the right axillary and left carotid artery, providing constant perfusion to the brain during arch surgery and minimally reducing cerebral ischemic time; likewise, we associated it with profound hypothermia (< 28 °C) that allowed an acceptable alteration of the internal environment, less bleeding and greater neuroprotection. It is known that on average the Cerebral Oxygen Metabolic Rate (COMO2) decreases 7% for every 1 °C reduction in temperature from 37 °C, allowing us to perform the surgical procedure safely until 45′–55′ before the occurrence of the significant neurological and multisystem side effects [7].

Recent studies describe a lower incidence of ischemic brain lesions demonstrated by magnetic resonance imaging with the use of TCADHCP-AB in relation to the same technique with moderate hypothermia (*p* < 0.01) [7]; however, others describe an incidence of 19.1% of transient ischemic attacks (OR 1.88, *p* = 0.23) when this strategy exceeds 25′–30′ associated with a survival of 61.8% at 10 years (57.8–65.8%, 95% CI, *p* < 0.05) [8].

Another effective method for adequate monitoring of cerebral perfusion in TCADHCP-AB is continuous cerebral oximetry (NIRS), used in more than 65% of the hospital institutions that perform this type of surgery, and allows the capture of regional oxygen saturation of the tissues corresponding to the perfusion territories of the anterior and middle cerebral arteries. Its main advantage is that it is not affected by the degree of anesthetic depth or by hypothermia, and in the case of an acute brain injury, its value is directly related to the COMO2 and decreases proportionally to the severity of the injury [6].

From a hemodynamic and flowmetric point of view, open surgical reconstruction of the aortic arch presents serious advantages over endovascular and hybrid, with 100–200% less pressure drop in regions of supra-aortic vessels with a uniformly distributed flow and with a nominal wall shear stress of 417 dyne/cm^2^. This fact has still demonstrated the hemodynamic supremacy of open aortic arch correction over the other options [9].

The repair of an ATAA with compromised aortic arch that respects the anatomical and hemodynamic principles described, with TCADHCP-AB support and optimal NIRS monitoring, should lead to low morbidity and mortality rates (< 25%) [7]. However, disorders of the internal environment that produce profound hypothermias, perioperative bleeding, and systemic/cerebral ischemia time are the main predictors of postoperative survival in the short and long term.

Current evidence describes that the use of TCADHCP-AB in the context of aortic arch surgery in centers with high volume of patients, survival to the first year is 89% (79–94%, 95% CI, *p* < 0.05), 78% at 5 years (66–86%, 95% CI, *p* < 0.05) and 73% at 10 years (59–82%, 95% CI, *p* < 0.05) [10]. Despite the aforementioned findings, our case is added to the list of the first to be performed at our hospital headquarters, and we are aware that evaluating survival in the medium and long term will be the subject of future research.

Recently, various combined endovascular treatments for ATA and aortic arch pathologies have been disseminated; however, they were only evaluated in isolated cases, not showing great benefits and presenting serious risks (stroke, endoleaks, myocardial infarction, embolism, etc.…) over standard open treatment [6].

## Conclusions

With the advent of new anesthetic and neuroprotection techniques, perioperative imaging protocols, advanced hemodynamic monitoring, and invaluable advances in perfusion and Extracorporeal Circulation (EC) with circulatory arrest, they have made this surgical challenge a valuable tool for today's cardiovascular surgeon. Despite the aforementioned, hybrid therapy has been gaining territory in this segment of the aorta, and its main advantage is the non-use of hypothermic brain perfusion strategies, which, as we know, is associated with a range of perioperative complications that cast a shadow on the prognosis of the patient [1]. As long as the safety of these novel techniques is not proven, the surgical territory of the aortic arch is today called “The last frontier of cardiac surgery”.

## Data Availability

Data sharing is not applicable to this article as no datasets were generated or analyzed during the current study.

## Abbreviations

ATA: Ascending Thoracic Aorta
ATAA: Ascending Thoracic Aortic Aneurysms
EC: Extracorporeal Circulation
TCADHCP-AB: Total Circulatory Arrest with Deep Hypothermic Cerebral Perfusion-Antegrade Bilateral
TE: Transthoracic Echocardiogram
COMO2: Cerebral Oxygen Metabolic Rate
RCA: Right Coronary Artery
LCA: Left Coronary Artery
BCT: Brachiocephalic-Trunk
RSA: Right Subclavian Artery
LSA: Left Subclavian Artery
RCa.A: Right Carotid Artery
LCa.A: Left Carotid Artery
DTA: Descending Thoracic Aorta
